# Formation and Evolution of Nanoscale Calcium Phosphate Precursors under Biomimetic Conditions

**DOI:** 10.1021/acs.analchem.1c01561

**Published:** 2021-07-12

**Authors:** Ludovica M. Epasto, Tristan Georges, Albina Selimović, Jean-Michel Guigner, Thierry Azaïs, Dennis Kurzbach

**Affiliations:** † Faculty of Chemistry, Institute of Biological Chemistry, University Vienna, Währinger Str. 38, 1090 Vienna, Austria; ‡ Sorbonne Université, CNRS, Laboratoire de Chimie de la Matière Condensée de Paris (LCMCP), 4, Place Jussieu, F-75005 Paris, France; § Institut de Minéralogie et Physique des Milieux Condensés (IMPMC), Sorbonne Université, 4, Place Jussieu, F-75005 Paris, France

## Abstract

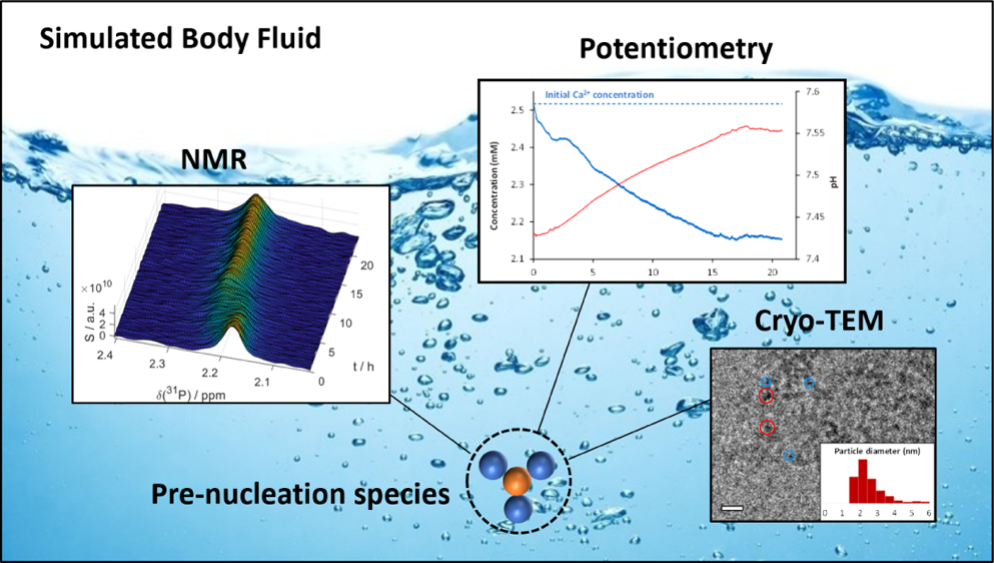

Simulated body fluids (SBFs) that mimic human blood plasma are widely used media for *in vitro* studies in an extensive array of research fields, from biomineralization to surface and corrosion sciences. We show that these solutions undergo dynamic nanoscopic conformational rearrangements on the timescale of minutes to hours, even though they are commonly considered stable or metastable. In particular, we find and characterize nanoscale inhomogeneities made of calcium phosphate (CaP) aggregates that emerge from homogeneous SBFs within a few hours and evolve into prenucleation species (PNS) that act as precursors in CaP crystallization processes. These ionic clusters consist of ∼2 nm large spherical building units that can aggregate into suprastructures with sizes of over 200 nm. We show that the residence times of phosphate ions in the PNS depend critically on the total PNS surface. These findings are particularly relevant for understanding nonclassical crystallization phenomena, in which PNS are assumed to act as building blocks for the final crystal structure.

## Introduction

Biomineralization is defined as the ability of living organisms to produce mineral phases embedded mostly in calcified tissues such as bone and teeth of vertebrates.^1−3^ Beyond its fundamental aspect, its understanding is also of imminent interest for developing nature-inspired materials with tailored properties such as improved bone implants^4^ or functional materials.^5,6^ In this context, simulated body fluids (SBFs) that mimic the ionic composition of human blood plasma^7,8^ are ubiquitous and used in a wide array of research fields, from the design of bone graft materials^9,10^ and tissue engineering^11,12^ to corrosion and biodegradation studies^13−15^ to bioinspired material design.^16,17^ In particular, SBF plays a crucial role as it is the most widespread biomimetic medium used to assess the bioactivity of materials.^18−20^ Notwithstanding its frequent use, the “nanostructure” of SBF remains unsolved to large degrees. A deeper understanding of the structural dynamics is yet essential due to the influence of solvent properties on mineralization and crystallization phenomena.^21,22^ Indeed, environmental conditions often determine the morphology of solid phases precipitating from solution.

In this regard, SBFs have gained particular attention as a result of the recent observation of so-called prenucleation species (PNS) and their involvement in the early onset of biomimetic calcium phosphate (CaP) precipitation (e.g., hydroxyapatite).^23,24^ These PNS are highly dynamic nanometric ionic clusters that spontaneously form in solution preceding the precipitation of solid crystalline phases.^25^ Today, PNS have been documented for CaP^21,23,26−29^ and carbonates,^30−33^ as well as for iron oxides.^34^ Interestingly, their involvement in the formation of biominerals challenges the long-standing nucleation-and-growth paradigm,^35,36^ which has stimulated an ongoing update of the existing crystallization theories.^26,37,38^

In addition, it has been suggested that the PNS structure and dynamics determine the crystallization path a material takes.^22^ Hence, a better understanding of PNS behavior in SBFs promises new avenues for the rational design of solid biomimetic ceramics via control over the initial reaction conditions that regulate the molecular architecture of the final material. However, the structure and dynamic behavior of PNS (in particular in SBF) remain poorly understood, and the factors that control the associated nucleation events remain unclear, leaving significant gaps in the current understanding of crystallization.^39,40^

Indeed, the highly dynamic behavior of SBFs and its ionic constituents is challenging to characterize experimentally from both, structural and dynamical, viewpoints. To overcome this bottleneck, we here present an integrative approach, including the first observation of PNS by real-time nuclear magnetic resonance (NMR) spectroscopy in solution combined with cryoelectron microscopy and calcium potentiometric measurements. We shed light on the constitution of SBFs at the nanoscale, revealing dynamical and structural inhomogeneities therein and thus providing a significant advancement in their understanding.

In particular, we show how SBF undergoes defined and irreversible molecular rearrangements with time, challenging the long-standing notion of its (meta)stability.^23,41,42^ We find that nanometric CaP clusters with diameters of ca. 2 nm form within 5 h after preparing the fresh SBF. We identify these clusters as previously described CaP-based PNS.^23^ Subsequently, these PNS can aggregate to form soluble suprastructures of several hundreds of nanometers in size that might precede the precipitation of solid CaP. We find calcium triphosphate units to be the basic units of the PNS. Furthermore, we characterize the exchange kinetics of phosphates between PNS-bound and free states, demonstrating that the frequency of exchange depends critically on the quantity of PNS in solution.

## Experimental Section

### Sample Preparation

Modified SBFs (mSBFs) were prepared following the protocol by Oyane et al.^43,44^ In brief, neat water was stirred at 37 °C, and the salts constituting mSBF were added successively to achieve concentrations comparable to those found in human blood plasma and in particular, a P_i_ concentration of 1 mM. After the addition of all the salts, the pH was adjusted to 7.4 using 1.0 M NaOH. After preparation, the mSBF was immediately transferred to an NMR tube, and 10% v/v D_2_O was added as the lock solvent.

### NMR Spectroscopy

All NMR data were acquired at 37 °C on a Bruker NEO NMR spectrometer operating at 11.7 T (500 MHz proton Larmor frequency) equipped with a 5 mm Bruker Prodigy BBO cryoprobe. For real-time detection, 1D ^31^P NMR spectra were acquired every 7.5 min by averaging 64 flame-ionization detectors (FIDs). For detection, 90° flip-angle pulses were applied with a length of 12 μs every 5 s. Prior to Fourier transformation, all data were zero filled and apodized using a Gaussian window function. Subsequent to Fourier transformation, all data were baseline-corrected. To extract signal intensities, the NMR signals were fitted to two Lorentzian functions using home-written scripts embedded in the MATLAB software package using the “fitNlorentzian” function.

For diffusion ordered spectroscopy (DOSY), the spectra were acquired using the dstebpgp3s pulse sequence^45^ embedded in the Bruker TopSpin 4 pulse sequence library. The spectra were acquired over 5h15 by averaging 150 FIDs. For detection, 90° flip-angle pulses were applied with a length of 12 μs every 10 s. A six-step *z*-gradient ramp was used with 2, 14, 25, 37, 48, and 60% of the maximum gradient strength.

Data were analyzed using the GNAT software package^46^ for MATLAB. Prior to Fourier transformation, all data were zero-filled and apodized using a Lorentzian window function. Subsequent to Fourier transformation, all data were baseline-corrected. Diffusion coefficients were extracted using monoexponential fits to the Stejskal–Tanner equation.^47^

### Calcium Ion Potentiometry

Calcium concentration and pH were monitored using a calcium-sensitive electrode (Ca-ISE, Metrohm) and a pH glass electrode (Unitrode, Metrohm) connected to a Titrando titration device (Metrohm) and analyzed with Tiamo software (Metrohm). The Ca-ISE was calibrated at room temperature using three modified SBF-like solutions with respective calcium concentrations of 0.1, 1, and 3 mM. These m-SBF-like solutions were synthetized by following the procedure of Oyane et al.^43,44^ omitting phosphate salts. For each solution, the potential (in millivolts) was measured and automatically fitted to the Nernst equation with a least-square algorithm. The pH electrode was calibrated using three buffered solutions at pH 4, pH 7, and pH 9 (Metrohm). Sample evolution was monitored for 24 h recording potential every 10 s under gentle stirring.

### Cryoelectron Microscopy and XEDS

m-SBF solutions were vitrified at specific time points after preparation (1, 5.5, and 23 h). The morphology and the size of nanoparticles were determined from cryotransmission electron microscopy (cryo-TEM) images. A drop (3 μL) of solution was deposited on “quantifoil” (Quantifoil Micro Tools GmbH, Germany) carbon membrane grids. The excess of the liquid on the grid was absorbed with a filter paper, and the grid was quench-frozen quickly in liquid ethane to form a thin vitreous ice film. The samples were transferred in the microscope and observed at low temperature (−180 °C). Cryo-TEM images were recorded on ultrascan 1000, 2k × 2k CCD camera (Gatan, USA), using a LaB_6_ JEOL JEM2100 (JEOL, Japan) cryomicroscope operating at 200 kV with a JEOL low-dose system (Minimum Dose System, MDS) to protect the thin ice film from any irradiation before imaging and reduce the irradiation during the image capture.

X-ray energy-dispersive spectra (XEDS) characterizing the elemental composition of the samples have been recorded with a JEOL (Japan) XEDS detector with 140 eV resolution using a JEOL (Japan) 2100F, field-emission gun instrument operating at 200 kV under cryo-conditions. Images were recorded on an UltraScan 4000 Gatan (USA) camera with a 4096 × 4096 charge-coupled device.

## Results and Discussion

In the following, we will first describe the formation and internal dynamics of PNS in SBFs by employing real-time NMR. Second, we describe the PNS constitution by means of transmission electron microscopy (TEM) and potentiometric experiments.

We want to stress that we herein collectively term all CaP aggregates that occur in solution during the early onset of CaP precipitation, for the sake of generality, prenucleation species or “PNS”. This notion should here be understood as neutral, that is, as not referring to any nucleation or crystallization theory.^26,37−39^

### Real-Time NMR of the Evolving SBF

We initially observed the formation and evolution of PNS by real-time NMR of the modified SBF^43,44^ (mSBF which provides improved pH stability over neat SBF). Capitalizing on the strong dependence of the chemical shift of phosphate ions on their local environment, these experiments could distinguish the signals of phosphates bound in a PNS from free phosphates in solution. They revealed that PNS form within 5 h after preparing fresh mSBF and undergo continuous changes throughout a 15 h period.

**Figure 1:**
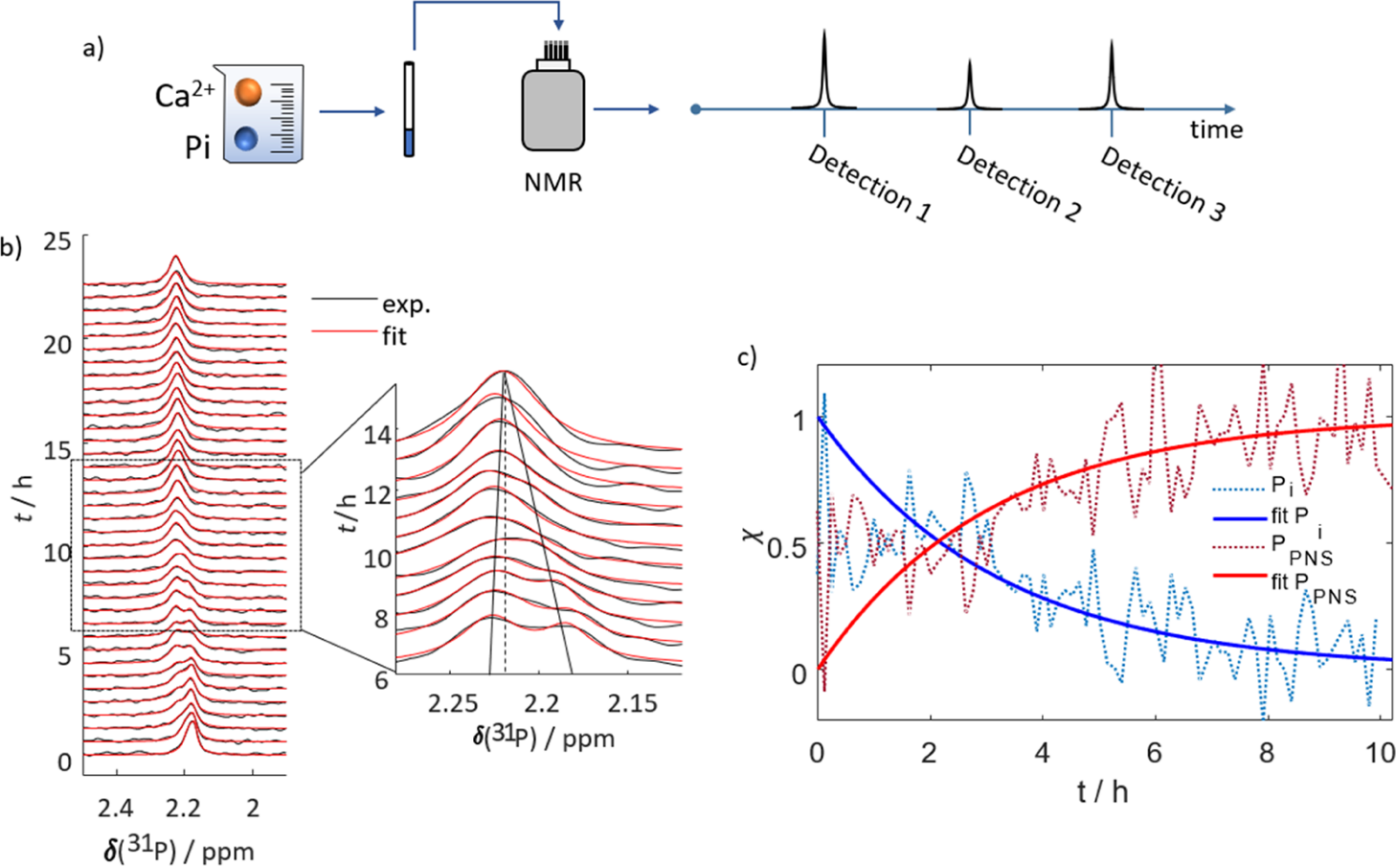
Monitoring of PNS formation in mSBF through real-time NMR. (a) Scheme of experimental workflow. The evolution of PNS at 37 °C in mSBF is traced by consecutive detections of ^31^P spectra. (b) ^31^P NMR signals detected for mSBF over a period of 24 h (black) and corresponding fits to two Lorentzian functions (red). Initially, only a single resonance can be observed. After ∼5 h, a second peak appears. Between 8 and 10 h, the first and second peaks merge, forming a single signal. The insert highlights the transition period during which the two signals merge. The resulting single line resonates at a frequency between those of the precursor signals. At *t* > 15 h, again, only a single line is visible. Note that the spectra are normalized. (c) Time-dependence of the fractional contribution χ of the two lines to the overall spectrum. The contribution of free phosphate is shown in blue and that of PNS-bound phosphate in red. The dotted lines indicate the experimental data, while the solids lines represent fits of the experimental data to monoexponential functions. After 10 h, the signals merged and the two contributions could not be separated anymore.

We recorded a series of ^31^P NMR spectra, as outlined in Figure 1a, starting immediately after the sample preparation. Figure 1b shows the resulting spectra (black lines) and superimposed fits (red lines) to two Lorentzian functions that model the NMR line shape (see the Experimental Section for details). At *t* = 0, a single resonance at 2.18 ppm is detectable, corresponding to free inorganic phosphate (P_i_) dissolved in mSBF similar in position and linewidth to the control experiment (Figure S1). After 3–5 h, a second broader peak appears at 2.23 ppm. We assign this signal to emerging PNS (*vide infra*) and denote the involved phosphates henceforth as P_PNS_. During this period, our fitting routine can quantitatively reproduce the spectra with only two lines. From 8 to 10 h after preparation, both signals are broadened compared to their initial line width and slowly approach each other.

After 10 h, the signals merge, and only a single line remains at a chemical shift of 2.21 ppm (insert Figure 1b). Notably, the total signal intensity is constant throughout the detection period (up to 30 h), indicating that CaP does not precipitate from mSBF under our conditions (*T* = 37 °C; pH = 7.4) throughout the experiment.

Figure 1c shows the relative fractions χ of the two spectral components of P_i_ and P_PNS_ of the spectra during the initial period of evolution. The figure highlights how P_i_ is consumed to form P_PNS_.

### Diffusion-Ordered Spectroscopy Provides Evidence of PNS

To characterize the two different phosphate species further, we employed DOSY. It showed that the hydrodynamic radius of the PNS is ca. 1.7-fold larger than that of free P_i_.

**Figure 2:**
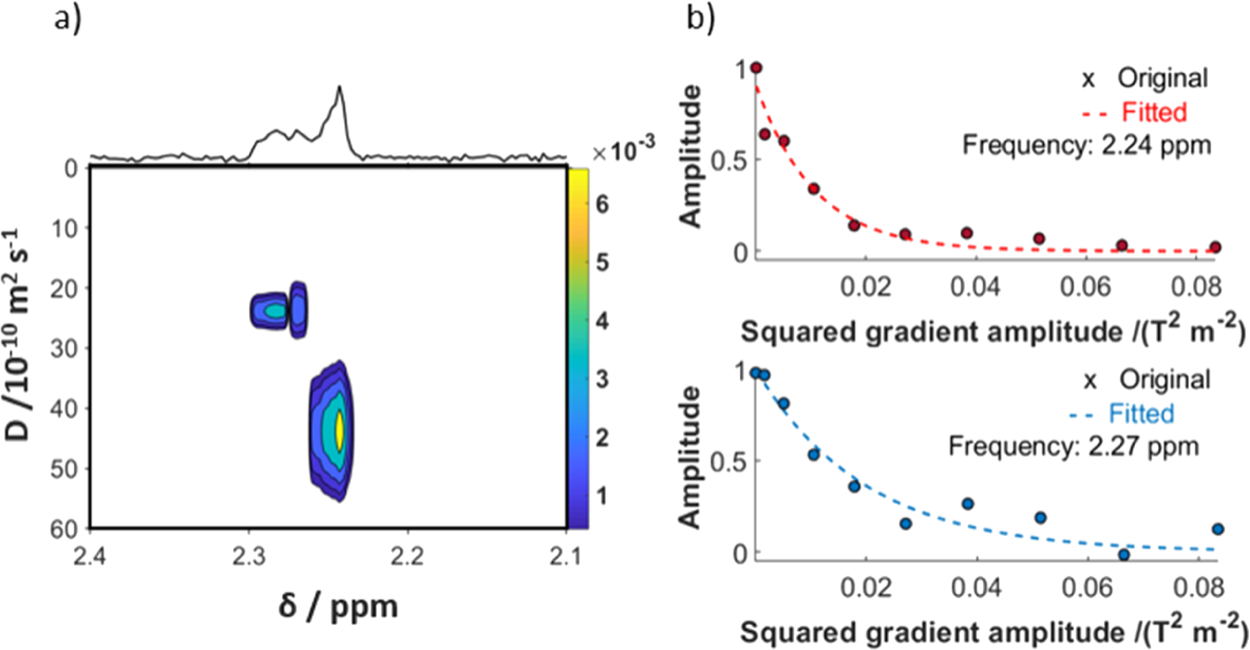
DOSY analysis of mSBF. (a) Mapped diffusion coefficients in dependence of the chemical shift. When both the P_i_ and the P_PNS_ signals, appear well-separated in the ^31^P NMR spectra, DOSY of the system allows one to discern two phosphate species via their diffusion coefficients. A slower diffusion coefficient is observed for the species underlying P_PNS_ (left) compared to the signal underlying P_i_ (right). The diffusion coefficients were ∼24 × 10^–10^ vs ∼43 × 10^–10^ m^2^ s^–1^, respectively. The data were acquired at 37 °C for 5 h at a phosphate concentration of 5 mM. (b) Monoexponential fits of the two DOSY profiles underlying the data in panel (a). The P_PNS_ signal decays slower with increasing gradient amplitude than the P_i_ signal.

Due to the low signal intensities, these experiments had to be acquired at a P_i_ concentration of 5 mM (a fivefold increase compared to neat mSBF). However, the results can be safely qualitatively interpreted as, similar to mSBF, two distinct resonances appear after 10 to 15 h of maturation (Figure S2). Figure 2 displays the DOSY analysis of modified mSBF acquired over a 5 h period (10–15 h after sample preparation). Notably, two distinct diffusion coefficients are evidenced. A coefficient of *D* = ∼24 × 10^–10^ m^2^ s^–1^ could be observed for the single P_PNS_ peak. The P_i_ signal yielded to a faster coefficient of ∼42 × 10^–10^ m^2^ s^–1^. Solving the Stokes–Einstein relation assuming spherical particles, we find that the average hydrodynamic radius *R*_h_ of P_PNS_ is ∼1.7-fold larger than that of P_i_, amounting to ∼1.0 and ∼0.6 nm, respectively.

Therefore, the volume of the detected particles is ca. fourfold larger than that of free P_i_. Hence, we deduce that the second signal appearing in the ^31^P spectra in Figure 1 indeed stems from phosphate units embedded in larger ionic clusters that, despite their size, remain in solution, that is, in a PNS.

### Theoretical Modeling of the Phosphate Exchange

To better understand the *R*_h_ variations and the real-time NMR data, we theoretically modeled our data assuming two-site chemical exchange. We found that the mSBF system passes from a slow to an intermediate to a fast exchange regime with growing PNS concentration. This circumstance causes the apparent spectral changes in the NMR time series.

**Figure 3:**
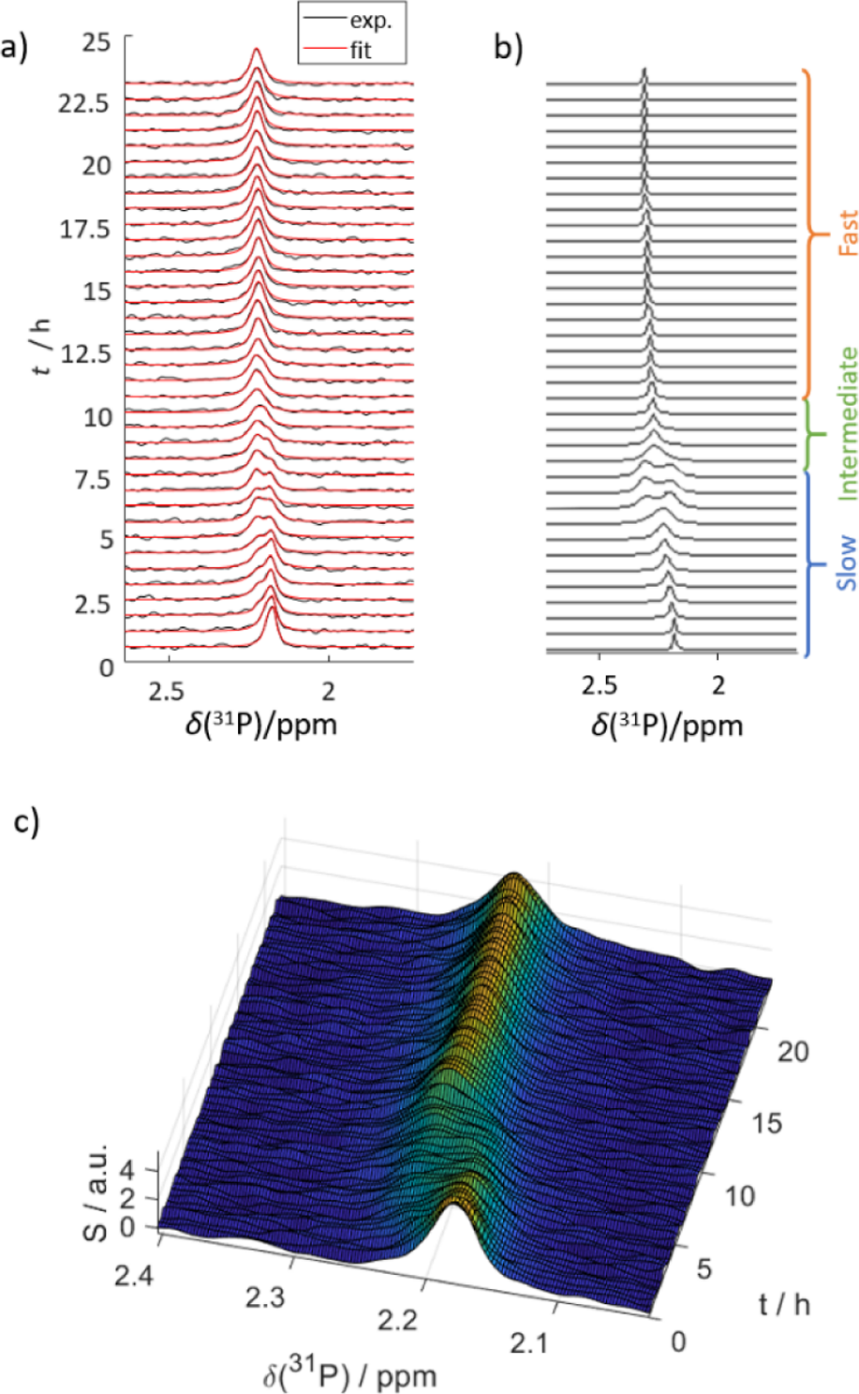
Comparison of (a) experimental results of mSBF evolution at 37 °C with (b) simulated data. The prediction was performed with a dynamic two-site exchange model, as described in the main text. In both, the second species appears at ca. 3–5 h, following the exchange path of the NMR-detected peak. The brackets indicate the different exchange regimes. (c) Non-normalized surface plot of the ^31^P NMR signal versus time. The representation illustrates that the signals are broadened and reduced in amplitude during the transition from slow to fast exchange.

To form PNS, it is necessary that the phosphates exchanges between the pool of free P_i_ in solution and the pool of newly bound species P_PNS_. This process influences the shape of the NMR spectra in Figures 3a (similar to 1b). The time series starts with a single P_i_ signal, followed by the emergence of a well-distinguishable P_PNS_ signal. Later, both signals first broaden and then merge into a single, again sharp, resonance. This behavior is typical for exchange-driven spectral coalescence.^48^Figure 3c illustrates how both signals are broadened and reduced in amplitude during the period of intermediate exchange.

We could model this behavior using the Norris equation^49^ for two-site exchange, which can be considered as a simplified version of the Carver–Richards equation^50^
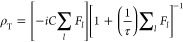
1a
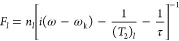
1bρ_T_ is the resulting spectrum, τ stands for the average inverse exchange rate *k*_ex_, and *n*_*l*_ is the fractional population of the *l*th site. ω is the resonance frequency and 1/(*T*_2_)_*l*_ is the line width of the *l*th species in the absence of exchange. *C* is an overall proportionality constant assumed here to be 1.

The exchange rate constant 1/τ was modeled by combining the classical collision theory Arrhenius law with a first-order kinetic equation
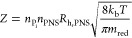
2a
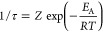
2b
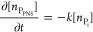
3where *n*_P_i__ and *n*_PNS_ indicate the concentrations of free and PNS-bound phosphates, respectively. *R*_h,PNS_ is the estimated radius of hydration of a PNS (assumed to be 1 nm, in line with the DOSY experiments). *k*_b_ is the Boltzmann constant, *T* is the temperature of the system, and *m*_red_ is the reduced PNS mass. *E*_A_ is the activation energy, and *R* is the gas constant. In eq 3, *k* is the kinetic constant of the conversion P_i_ → P_PNS_.

Combining eqs 1a–2a, we could reproduce the experimental data, as shown in Figure 3a,b. At this stage, the following understanding emerges of the exchange processes in mSBF:

Initially, aggregation of P_i_ and Ca^2+^ ions leads to CaP-PNS formation and the emergence of a corresponding second signal. At this time, the system is in slow exchange as the number of PNS is still small, compared to free P_i_, such that only a small “exchange surface” is available and a small number of encounters (cf. eqs 2a and 2b; the prefactor *n*_PNS_ is small, leading to slow exchange rates 1/τ). After 5 h, the two peaks broaden as the system undergoes a transition into an intermediate exchange regime, where Δδ(^31^*P*) = 1/τ = *k*_ex_. In other words, the exchange surface grows together with the number of PNS, *n*_PNS_. Finally, the two peaks merge after ca. 15 h, with a chemical shift averaged between the two initial lines (cf. Figure 1b). For this case, the model calculations indicate that the phosphates experience a fast exchange between both states, that is, Δδ(^31^*P*) < 1/τ.

The real-time NMR data, diffusion measurements, and theoretical predictions thus suggest a process in which (i) PNS forms in mSBF spontaneously after its preparation and continues to emerge over a time of >15 h, (ii) the diameter of the PNS detected by solution-state NMR is on the order of 2 nm, and (iii) the exchange between freely dissolved and PNS-bound phosphates accelerates when the concentration of PNS grows.

During the slow exchange phase, the chemical shift difference between the two species amounts to 0.05 ppm, which corresponds to an exchange rate of *k*_ex_ < 10 Hz. Interestingly, earlier studies on CaP precipitation (dicalcium phosphate dehydrate )^51^ under nonbiomimetic conditions (pH 8 and ion concentrations >10 mM) reported much higher limits of *k*_ex_ < 250 to 350 Hz, highlighting the importance of environmental conditions for crystallization studies.

### Cryo-TEM Analysis Reveals PNS Suprastructures

**Figure 4:**
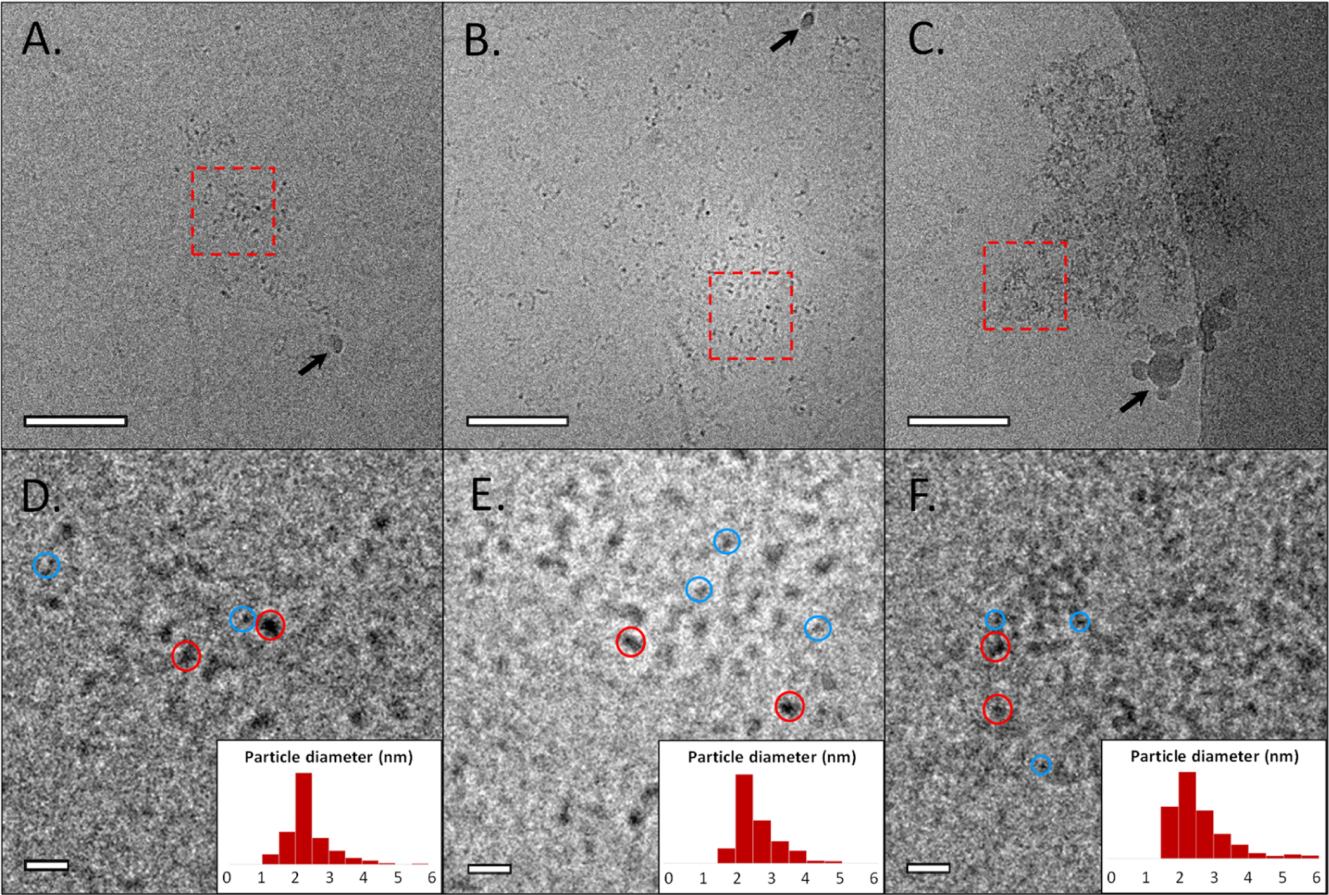
Cryo-TEM observations of mSBF solution at different time points—(A) 1 h, (B) 5.5 h, and (C) 23 h after mSBF preparation, respectively. (D–F) Zoom of the corresponding zones delimited by the red squares in (A–C). Insert displays size evaluation of individual PNS. Blue and red circles give an example of smaller and bigger PNS, respectively. The black arrows indicate the presence of polluting agents, due to the freezing process. Scale bar (A–C) = 50 nm. Scale bar (D–F) = 10 nm.

To further analyze the PNS structure, we performed cryo-TEM observations. Data were acquired 1, 5.5, and 23 h after sample preparation, following the exchange regimes derived by NMR (Figures 4 and S3). After 1 h, we observed the appearance of the first PNS (Figure 4a). The number of PNS visibly increased after 5.5 h (Figure 4b,c), supporting the time-dependent emergence of PNS in the mSBF. After 23 h of maturation, some PNS are found to aggregate into higher-order structures of several hundreds of nanometers including typically ∼100 to ∼400 PNS (Figure 4c). In addition, free PNS were also observed to coexist in solution (Figure S3).

Higher magnification allows the evaluation of PNS size and distribution (Figure 4d–f). After 1 and 5.5 h, individual PNS with sizes ranging from 1.5 to 3.5 nm were observed in good agreement with the DOSY NMR data. After 23 h, PNS aggregates were found constituted by individual spherical units again with diameters of 1.5–3.5 nm. In addition, bigger units of 4–6 nm in size were found to coexist at this time of maturation (red circles vs blue circles in Figure 4d–f).

The observed aggregates and clusters remain in solution in the mSBF for an extended period before CaP precipitation takes place. Hence, these structures likely define the starting point of the pathway CaP takes toward its solid crystalline state.^23,24,27^

Note that solution-state NMR is limited to aggregates of a few nanometers due to line-broadening effects for bigger molecular assemblies. It is very likely that the detected P_PNS_ resonance through ^31^P NMR species do not reflect all PNS observed by cryo-TEM. Larger species might not be detected. The determined *R*_h_ of ∼1 nm through DOSY NMR corroborates this assumption.

### EDXS Analysis Reveals Phosphate-Rich PNS

**Figure 5:**
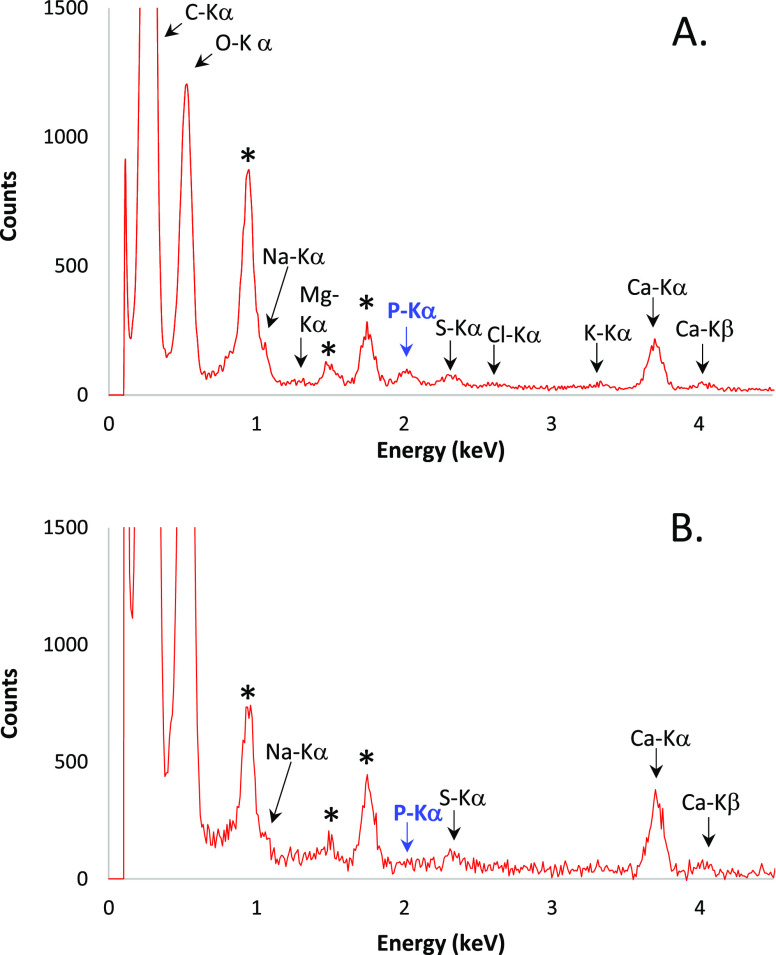
EDXS analysis of the mSBF performed 23 h after preparation. (a) Selected zone contains a large amount of PNS. (b) Selected zone does not contain a significant amount of PNS (Figure S4). The electron beam area is ∼1 μm^2^. Black stars at 0.93, 1.49, and 1.74 keV indicate Cu-Lα, Al-Kα, and Si-Kα peaks, respectively, corresponding to the grid, its plasma treatment, or pollution.

We employed energy-dispersive X-ray spectroscopy (EDXS) of mSBF 23 h after preparation to determine the PNS composition after complete equilibration. In the electron micrograph, we selected a region of ∼1 μm^2^ containing a large PNS aggregate (Figure 5a) and another region devoid of any PNS (Figure 5b) as a negative control.

The Ca Kα and Ca Kβ peaks are both visible in the absence and presence of PNS. In opposition, the P Kα peak is only visible in the presence of PNS, indicating the abundance of phosphorous atoms within the ionic clusters. Interestingly, S Kα and Na Lα signals (markers of sulfate and Na^+^), of comparable intensity to the P Kα peak, are present in both cases (Figure 5a,b).

Thus, the EDXS results suggest that the mSBF, once equilibrated, is depleted in free P_i_, while most phosphate ions are localized within the PNS units. These data are consistent with the ^31^P NMR data, in which the detected fraction of free P_i_ is very small at *t* > 15 h.

### Ca^2+^ Potentiometry Reveals the PNS Ca/P Ratio

To further corroborate the NMR-derived kinetics and determine the protonation state of the phosphate units, we potentiometrically determined the calcium ion concentration over a period of 21 h following mSBF preparation.

**Figure 6:**
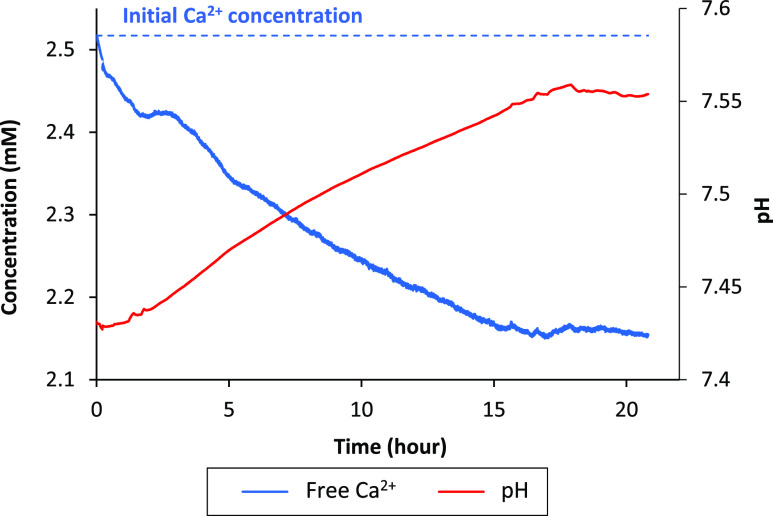
Evolution of free Ca^2+^ concentration (blue curve) and pH (red curve) of mSBF over time. Concentration curves were temperature-corrected to mitigate biases in apparent concentrations.

Figure 6 displays the time dependence of the free Ca^2+^ ion concentration throughout our experiments. After mSBF preparation, the concentration continuously decreases until it reaches a steady state at *t* > 16 h. This decrease results from the uptake of Ca^2+^ ions by the PNS (in line with the NMR and TEM data). The proportion of bound calcium ions after ca. 16 h amounts to 14% of the initial Ca^2+^ concentration, that is, the free ion concentration drops from 2.5 to 2.15 mM (Figure 6). In return, the amount of bound Ca^2+^ is *ca.* 0.35 mM (Figure S5). Moreover, from the NMR experiments and EDXS analysis, we can estimate the amount of P_PNS_ after 21 h to *ca*. 1 mM considering that the majority of phosphate ions are embedded in PNS. Thus, we determine that one Ca^2+^ ion interacts with 2.8 (∼3) P_i_, forming PNS as “calcium triphosphate units”.

Interestingly, we also observed a small pH variation strongly correlated with the free Ca^2+^ species (Figure 6). The pH increases from 7.42 to 7.56 (ΔpH = 0.14), reaching a plateau after 16 h similar to the concentration of free Ca^2+^.

This observation is important as the pH influences the phosphate speciation. The pH values suggest that PNS tend to accommodate both HPO_4_^2–^ and H_2_PO_4_^–^ ions but with a higher proportion of the latter ([H_2_PO_4_^–^]/[HPO_4_^2–^] = 0.36) (Figure S5).

Considering relative contributions of bound H_2_PO_4_^–^ and HPO_4_^2–^ of 36% and 64%, respectively, the PNS basic unit amounts to average nominal composition of [Ca(H_2_PO_4_)_1.04_(HPO_4_)_1.76_]_*n*_^2.56–^ (not considering coordinated water molecules), very similar to the calcium triphosphate units proposed by Habraken et al.^27^

## Conclusions

Our data show that the mSBF is a highly dynamic system that undergoes a continuous transformation after its preparation. It can be considered as stable or metastable only after an extended equilibration period of >24 h. Priorly, the dynamic emergence of CaP PNS continuously changes the nanoscopic constitution of the solution.

**Figure 7:**
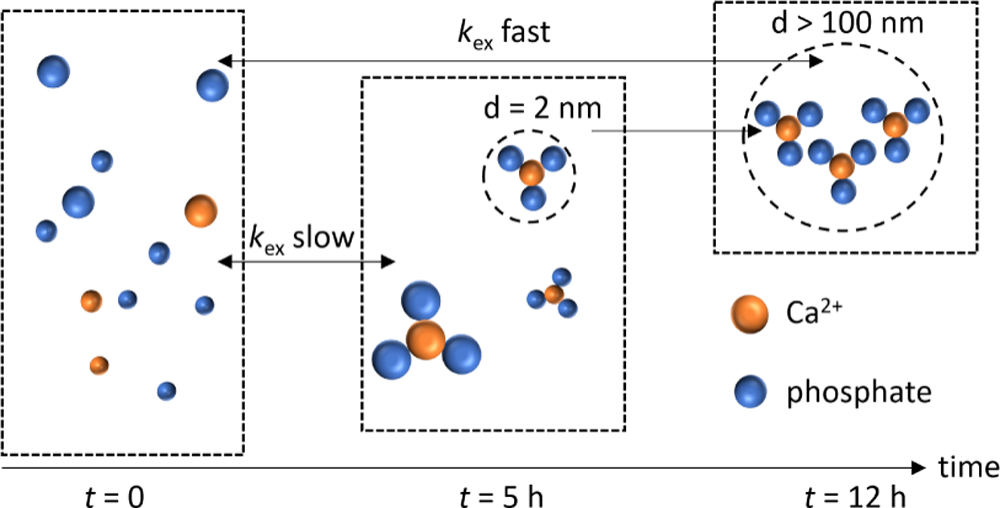
Scheme summarizing the PNS evolution in mSBF. After preparation, the ions are freely dispersed in solution. After 5 h, they aggregate to from CaP species with a diameter of ca. 2–3 nm. The exchange rate of phosphates between free and bound states is slow (on the NMR timescale) at this stage. After 12 h, the CaP clusters can aggregate to form suprastructures of more than 100 nm in size, where individual PNS are still present. The phosphate exchange is fast at this stage. The PNS are constituted by three phosphate units per Ca^2+^ ion.

Figure 7 summarizes the picture we developed of PNS in the mSBF from individual ions to small clusters to larger suprastructures. Initially, PNS solute species of ∼2 nm in diameter form right after mSBF preparation and experience accelerating phosphate exchange with maturation time. After equilibration, mSBF is found to be depleted of free phosphate as PNS form with an estimated Ca/P molar ratio of 1/3.

Given its widespread use, the understanding of SBF and PNS dispersed therein might be useful in a wide array of research fields. Among other examples, the investigation of nonclassical crystallization pathways in the field of biomineralization or the development of novel bone-graft replacements relies heavily on SBF use.

The combination of NMR, Cryo-TEM, and Ca^2+^ titration thereby provides the possibility of tracing the transformation of P_i_ into P_PNS_ in real time and quantify the size and composition of PNS. Therefore, the presented integrative methodology might open new avenues for spatiotemporally resolved data sets that enable characterization of nanoscale inhomogeneities in solution and associated crystallization events at an improved level of detail.

## Supporting Information Available

Reference NMR experiments, supplementary cryo-TEM images, and supplementary potentiometry experiments (PDF)
