# Supplementary material for: Formation and Evolution of Nanoscale Calcium Phosphate Precursors under Biomimetic Conditions
Source: Anal Chem. 2021 Jul 12;93(29):10204–11. doi: 10.1021/acs.analchem.1c01561 (PMC8319911; doi:10.1021/acs.analchem.1c01561)
Supplement: Supplementary file 1 — ac1c01561_si_001.pdf [file ac1c01561_si_001.pdf]

SUPPORTING INFORMATION

Formation and evolution of nanoscale calcium phosphate precursors under biomimetic conditions

Ludovica M. Epasto[#,$], Tristan Georges[+,$], Albina Selimović[#], Jean-Michel Guigner,[†] Thierry Azaïs[+,], Dennis Kurzbach[#,*]*

[#]University Vienna, Faculty of Chemistry, Institute of Biological Chemistry, Währinger Str. 38, 1090, Vienna, Austria.

[+]Sorbonne Université, CNRS, Laboratoire de Chimie de la Matière Condensée de Paris (LCMCP), 4, place Jussieu, F-75005 Paris, France

[†] Sorbonne Université, Institut de Minéralogie et Physique des Milieux Condensés (IMPMC), 4, place Jussieu, F-75005 Paris, France

[$] These authors contributed equally

**Corresponding authors: DK: dennis.kurzbach@univie.ac.at; TA: thierry.azais@sorbonne-universite.fr*

Table of contents

Page

S2. Figure S1: 1mM P_i + NaCl negative control;

S3. Figure S2: 5mM P_i in mSBF evolution;

S4. Figure S3: Cryo-TEM of PNS in mSBF ;

S5. Figure S4: Cryo-TEM of areas analyzed with EDX;

S6. Figure S5: bound-Ca^{2+} calculation.

Figure S1

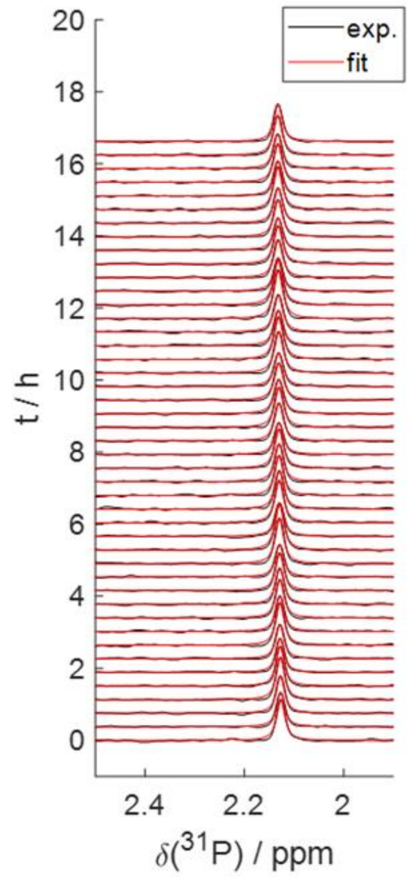

Figure S1. Negative control experiment of 1 mM Pi solution in which all salts (except Pi) from mSBF have been replaced by NaCl at equivalent concentrations to keep ionic strength constant (in HEPES buffer at pH 7.4). The ^{31}P NMR time traces are constant over time and exhibit one single resonance of similar position and line width to mSBF at t = 0. This control allows us to assign unambiguously the 2.18 ppm ^{31}P resonance of mSBF to free Pi.

Please note that mSBF solution without Pi cannot be used as a control because of calcite precipitation in these conditions (see Oyane *et al. J. Biomed. Res. A* **2003**, 64A, 339-348).

Figure S2

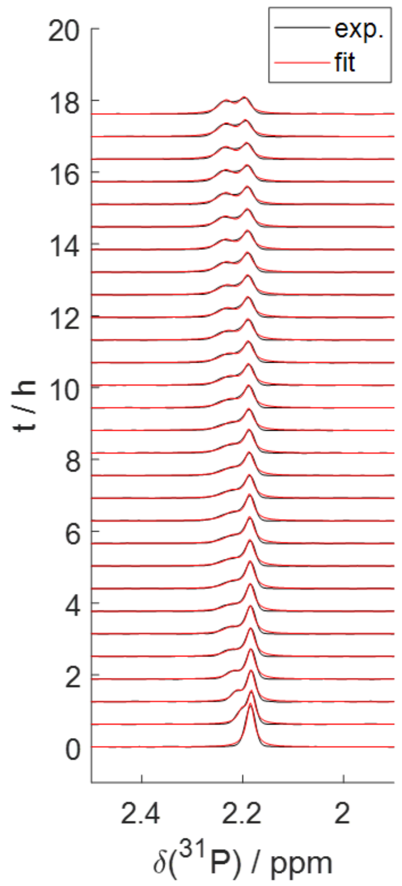

Figure S2. ^{31}P NMR time traces of 5 mM P_i in mSBF as used in the DOSY experiments. The evolution is qualitatively the same as in the case of neat mSBF. However, the evolution is slowed down due to the excess in phosphate, which enables the detection of longer DOSY experiments in the slow exchange regime.

Figure S3

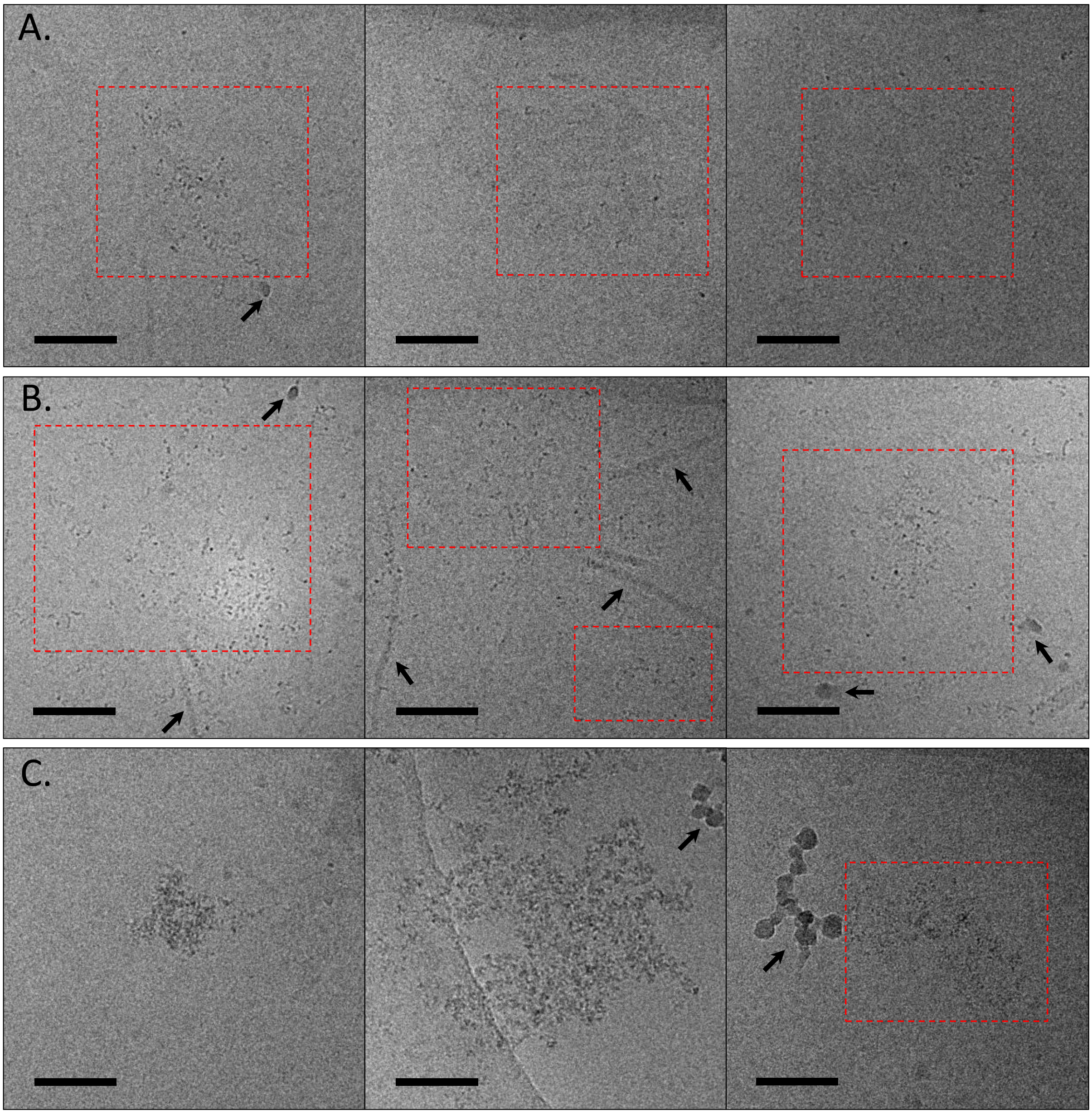

Figure S3. Evolution of PNS formation in mSBF over time revealed through cryo-TEM. (**a**) 1h, (**b**) 5.5h and (**c**) 23h after preparation. Scale bars = 50 nm. Red rectangles surround PNS units, black arrows point pollutions due to the freezing process, the plasma treatment of the grid or the solution container

Figure S4

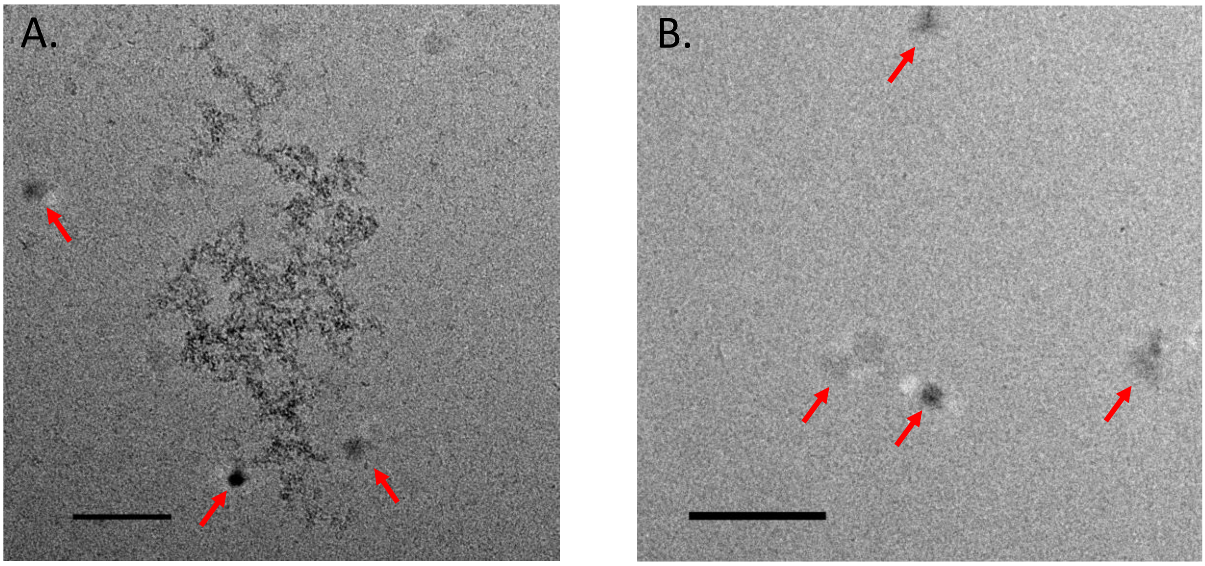

Figure S4. Cryo-TEM micrographs corresponding to the zones analyzed through EDX. (A) zone containing PNS. (**B**) zone empty of PNS (**B.**). Scalebar = 100 nm. Red arrows exhibit pollutions due to the freezing process and the plasma treatment of the grid.

Figure S5

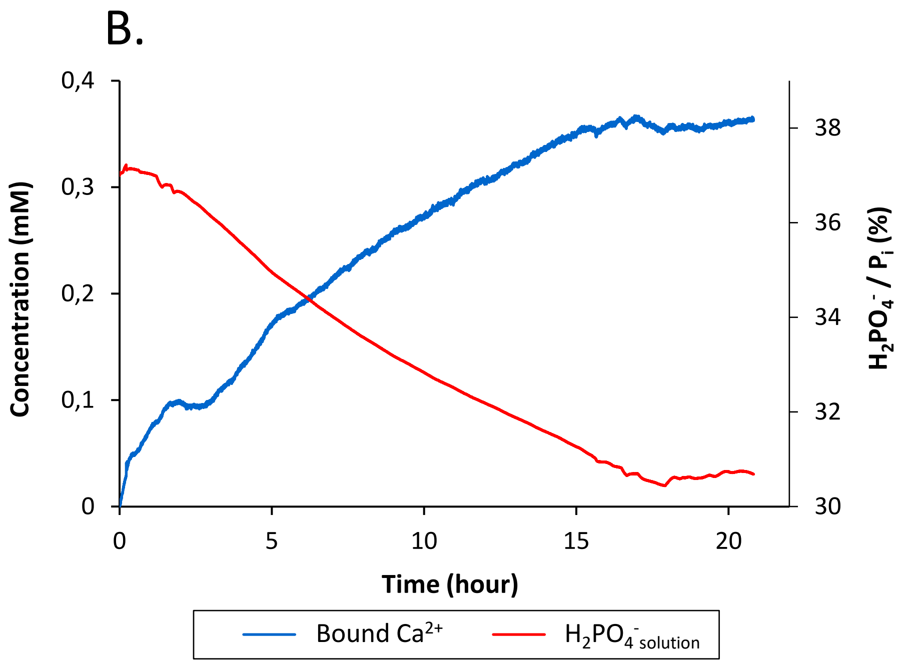

Figure S5. Calculation of bound-Ca^{2+} involved in PNS (blue curve) and $H_2PO_4^-$ concentration (red curve) in mSBF over time.
